# α-Synuclein seed amplification assay positivity beyond synucleinopathies

**DOI:** 10.1016/j.ebiom.2025.105925

**Published:** 2025-09-20

**Authors:** Ivan Martinez-Valbuena, Sarah Fullam, Sean O’Dowd, M. Carmela Tartaglia, Gabor G. Kovacs

**Affiliations:** a Tanz Centre for Research in Neurodegenerative Diseases, University of Toronto, Toronto, Canada; b Krembil Brain Institute, University Health Network, Toronto, Ontario, M5T 0S8, Canada; c Rossy Centre for PSP, Toronto Western Hospital, Toronto, Ontario, M5T 2S8, Canada; d Department of Neurology, Tallaght University Hospital, Dublin 24, Ireland; e Academic Unit of Neurology, Trinity College Dublin, Ireland; f Tallaght University Hospital Institute of Memory and Cognition, Ireland; g Department of Neurology, University of Toronto, Toronto, Ontario, M5T 1A8, Canada; h Edmond J. Safra Programme in Parkinson’s Disease and the Morton and Gloria Shulman Movement Disorders Clinic, Toronto Western Hospital, Toronto, Ontario, M5T 2S8, Canada; i Department of Laboratory Medicine and Pathobiology, University of Toronto, Toronto, Canada; j Laboratory Medicine Programme, University Health Network, Toronto, Canada

**Keywords:** α-Synuclein, Seed amplification assays, Co-pathology, Alzheimer’s disease, Progressive supranuclear palsy, Precision medicine

## Abstract

Neurodegenerative diseases are increasingly recognized as complex disorders involving multiple protein pathologies, with α-synuclein frequently observed beyond classical synucleinopathies such as Parkinson’s disease and multiple system atrophy. Recent advances in seed amplification assays (SAAs) have enabled the highly sensitive and specific detection of misfolded α-synuclein *in vivo*, particularly in cerebrospinal fluid (CSF). This review focuses on CSF-based α-synuclein SAAs and their application in detecting co-pathology across non-synucleinopathies, including Alzheimer’s disease, progressive supranuclear palsy, corticobasal syndrome, idiopathic normal pressure hydrocephalus, and traumatic brain injury. Evidence indicates a role for α-synuclein in clinical heterogeneity and disease progression. Emerging diagnostic frameworks increasingly support integrating co-pathologies into classification and therapeutic strategies. Addressing key knowledge gaps, such as α-synuclein interactions with other protein pathologies, and current limitations of α-syn SAA, such as the lack of quantification of misfolded α-synuclein seeds, will refine precision medicine and improve outcomes for patients with neurodegenerative diseases.

**Search strategy and selection criteria**

We searched PubMed for studies published from the database’s inception to May 2025. Our aim was to identify key publications on α-synuclein seed amplification assays (α-syn SAA) applied to cerebrospinal fluid (CSF) from patients with non-synucleinopathies. Studies were excluded if they involved non-synucleinopathy cases solely to assess assay sensitivity or specificity relative to classical synucleinopathies.

The core search terms included: (“Alzheimer’s disease” OR “progressive supranuclear palsy” OR “corticobasal syndrome” OR “idiopathic normal pressure hydrocephalus” OR “traumatic brain injury”) AND (“seed amplification assays” OR “RT-QuIC”) AND biomarkers. Additional disease-specific terms were used to tailor searches for individual sections. No language restrictions were applied.

This review is not intended as a systematic review, but rather as a narrative synthesis informed by a targeted literature search and expert appraisal. The final reference list was curated based on relevance to the review’s objectives and selected through author consensus, rather than via systematic screening protocols.

## Introduction

α-Synuclein seed amplification assays (α-syn SAAs), such as real-time quaking-induced conversion (RT-QuIC) and protein misfolding cyclic amplification (PMCA), have revolutionized the diagnosis of Parkinson’s disease (PD) by detecting misfolded α-synuclein in living patients with exceptional sensitivity.^1^, ^2^, ^3^ These assays exploit the self-propagating nature of misfolded α-synuclein, amplifying its signal through thioflavin T (ThT) fluorescence to achieve reliable detection.^1^ However, their specificity is reduced when applied to other neurodegenerative conditions such as in Alzheimer’s disease (AD), progressive supranuclear palsy (PSP), and corticobasal syndrome (CBS) that are associated with other proteinopathies but may show frequent presence of α-synuclein as a co-pathology.^4^^,^^5^ This overlap, rather than diminishing the utility of SAAs, underscores the clinical relevance of detecting α-synuclein seeding in non-synucleinopathies, influencing disease progression and phenotypic expression.^6^^,^^7^ This review highlights the significance of detecting α-synuclein co-pathology *in vivo* using SAAs across a spectrum of neurodegenerative disorders, demonstrating its widespread clinical relevance beyond the diagnosis of synucleinopathies. The complex interplay of mixed proteinopathies underscores the urgent need for a multimodal approach that integrates fluid-based biomarkers with advanced neuroimaging techniques. This integrated approach promises to refine the biological classification of neurodegenerative diseases, ultimately advancing precision diagnostics and enabling the development of targeted therapeutic interventions.

## Lessons from neuropathology

Advances in detecting α-synuclein pathology through *in vivo* α-syn SAAs must be anchored in neuropathological studies, which define the biological basis of synucleinopathies, elucidate the role of co-pathologies, and reveal critical insights into the anatomical and structural diversity of α-synuclein aggregates.^7^ This section summarizes the neuropathological understanding of α-synucleinopathies, the prevalence and impact of co-pathologies, and the lessons learnt from postmortem analyses that inform the interpretation of *in vivo* findings.

α-Synucleinopathies are a subset of neurodegenerative diseases marked by the selective dysfunction and loss of neurons and synaptic connectivity, alongside the pathological accumulation of conformationally altered α-synuclein in neurons and/or glial cells. The term proteinopathy is used when there are abundant filamentous aggregates of misfolded proteins that follow typical patterns of cellular involvement and correlate with clinical phenotypes and neurodegeneration. In contrast, protein pathology refers more broadly to the presence of assembled proteins, as detected by histochemical or molecular techniques—such as immunohistochemistry, silver staining, electron microscopy, immunoblotting, or SAAs—without necessarily inferring a distinct clinicopathologic entity.^8^

When interpreting α-synuclein pathology in postmortem studies, several key factors must be considered: the clinical phenotype, the distribution and stage of neurodegeneration, the sequential anatomical involvement, the intracellular aggregation stage (i.e., pre-aggregates vs. fibrillar inclusions), and the presence of co-existing proteinopathies or vascular lesions. These must be contextualized within the framework of mixed pathologies, which are highly prevalent in ageing and neurodegeneration.^4^^,^^5^

Based on these concepts, current classification of synucleinopathies include clinical, morphological, and, most recently, structural aspects. Two major groups are distinguished: the neuron predominant forms showing Lewy body pathology (LBP) and those exhibiting predominant oligodendroglial inclusions (Papp-Lantos bodies) defining MSA. Lewy body diseases (LBD) are stratified based on the clinical phenotype as PD, PD with dementia (PDD), or Dementia with Lewy bodies (DLB). More recent studies have investigated structural aspects of α-synuclein. Cryogenic electron microscopy (cryo-EM) studies revealed uniform folds in LBD (PD, PDD, and DLB)^9^ that are distinct from the filament fold in typical MSA cases^10^ and from that in juvenile onset synucleinopathy (JOS).^11^ However, our recent study indicates that Lewy-MSA hybrid folds can also be generated leading to distinct neuronal synuclein pathology in atypical form of MSA.^12^ Moreover, SAAs using post-mortem brain homogenates revealed that specific reaction buffer conditions can effectively differentiate the seeding profiles characteristic of MSA from those associated with Lewy body disorders.^13^

Regarding LBD, one neuropathological approach applies staging of α-synuclein pathology according to Braak and colleagues,^14^ while another approach identified brainstem, limbic, and neocortical types of Lewy body disease.^15^ Importantly, Lewy bodies can also be detected incidentally in the olfactory bulb, brainstem, or amygdala in the absence of clinical symptoms. Further updates for the neuropathology diagnostic practice have been described to integrate these observations.^16^ It should be emphasized that most studies focus only on Lewy bodies, however, less appreciated disease-associated α-synuclein immunoreactivities in LBD in the brain, such as in astrocytes, oligodendrocytes, and ependyma, have been also described.^17^ Regarding MSA, the presence of α-synuclein immunoreactive argyrophilic oligodendroglial inclusions (Papp-Lantos bodies) is sufficient for the diagnosis of MSA^18^ and clinical subtypes such as MSA-P (parkinsonism dominant) and MSA-C (cerebellar symptom predominant) are defined with striatonigral or olivopontocerebellar predominance, respectively.

**Fig. 1:**
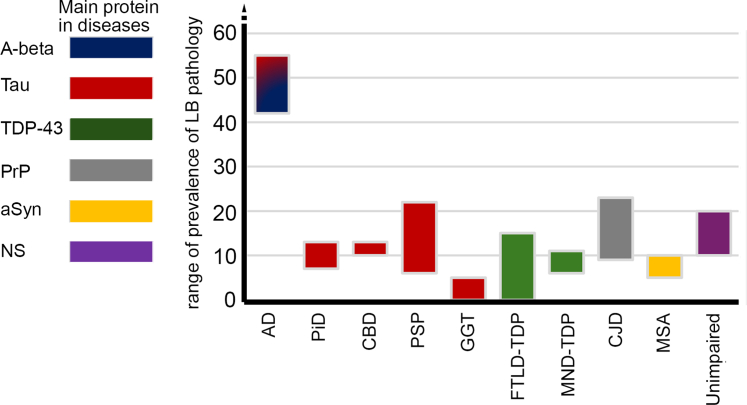
Range of prevalences of Lewy body (LB) pathology (Braak stage 3≥) reported in autopsy studies that used a systematic approach and evaluation. Representative ranges are reported for Alzheimer’s disease (AD),^22^^,^^23^ Pick’s disease (PiD),^23^^,^^24^ corticobasal degeneration (CBD),^23^^,^^24^ progressive supranuclear palsy (PSP),^23^^,^^24^ for globular glial tauopathies (GGT),^25^ frontotemporal lobar degeneration with tau or TDP-43 inclusions (FTLD-TDP, depending on whether sporadic or genetic),^23^^,^^24^ motor neuron disease with TDP-43 pathology (MND-TDP),^23^^,^^24^ Creutzfeldt-Jakob disease (CJD),^26^^,^^27^ multiple system atrophy (MSA),^23^^,^^28^ and cognitively unimpaired.^29^^,^^30^ On the left side colour coding of major neurodegenerative proteins are indicated. NS: not specified. A cautionary note is that the age groups in the different reports are not matched and studies on cognitively unimpaired represent older age categories and do not necessarily report lack of movement disorder.

LBD and MSA represent disease where α-synuclein pathology is consistently identified, while there are many conditions where they are detected inconsistently.^19^ The association of α-synuclein pathology with different neurodegenerative conditions can be discussed from two perspectives: when α-synuclein appears as co-pathology and when other neurodegenerative protein pathologies are observed in LBD and MSA.^20^^,^^21^ Indeed, a combination of pathological alterations is commonly observed in the brains of elderly individuals and in neurodegenerative diseases. However, due to the inconsistencies in the nomenclature and methodologies used (i.e., antibodies, criteria etc.) in various studies, and lack of a standardised classification of mixed pathologies it is difficult to decipher all details of the presence of α-synuclein as co-pathology. While it is difficult to dissect when LBD is the primary disorder or the co-pathology, literature data suggests that cases diagnosed as LBD on a neuropathology level have mostly limbic TDP-43 pathology and AD neuropathologic change as co-pathology. On the other hand, wide range of prevalences of LBP as co-pathology have been reported in various neurodegenerative proteinopathies (Fig. 1).^5^^,^^21^ Unfortunately, MSA pathology is usually not sought in the diagnostic screening for concomitant pathologies, in particular, that early steps of the disease might involve only the cerebellum.^31^ Interestingly, asymptomatic MSA cases have been identified in post-mortem studies of elderly individuals through α-synuclein immunostaining across multiple anatomical regions.^32^

Based on neuropathological studies, the following points need to be considered when we detect misfolded α−synuclein:

1. Caution is needed to consider α-synucleinopathy as a disease based on the mere detection of seeding of misfolded α-synuclein and a descriptive (presence or absence) approach is recommended since it is not necessarily associated with neuronal degeneration and disease.^19^
2. We need more observations whether distinct cytopathologies are associated with different protein structure and seeding.^12^^,^^33^
3. The combination of α-synuclein with any other misfolded protein pathology can be a foundation for disease diversity that might reflect distinct “biological” associations, warranting unique approaches to clinical diagnosis^19^ and eventually the interpretation of SAAs.
4. The reported frequency of cases with LBD as co-pathology varies considerably across neuropathological studies, therefore, it is always important to focus on how the inclusion criteria of cohorts were defined, whether the methods to evaluate misfolded α-synuclein were standardised, and whether all factors, that for example can influence SAAs, were excluded or considered for the interpretation. Some of these considerations might explain why SAA studies may report different prevalences.

## α-Synuclein biomarkers and their clinical relevance

As previously mentioned, large-scale neuropathological studies have paved the way and highlighted the substantial prevalence of α-synuclein protein pathology across non-synucleinopathies.^5^ However, the terminal neuropathological landscape may not fully depict the dynamic interplay of multiple proteinopathies throughout the disease course and the advent of *in vivo* biomarkers offers a transformative opportunity for real-time analysis of the misfolding of these proteins during life.^6^ In this section, we summarize recent advances in understanding the clinical significance of misfolded α-synuclein detected in cerebrospinal fluid (CSF) - using α-syn SAAs- across four distinct neurological conditions.

### Alzheimer’s disease (AD)

Given that AD is the most prevalent neurodegenerative disease worldwide^34^ and that a high rate of α-synuclein protein pathology is observed in AD brains,^5^ numerous studies in recent years have aimed to determine the prevalence and impact of α-synuclein co-pathology, as determined by α-syn SAA, in patients with AD^6^^,^^7^^,^^35^, ^36^, ^37^, ^38^ (Table 1). These studies have reported varying prevalence rates of α-synuclein co-pathology, ranging from 12 to 45%,^6^^,^^7^^,^^35^^,^^37^ which are often lower than those reported in analogous neuropathological studies.^5^ One critical aspect to consider when interpreting these percentages is the sensitivity of α-syn SAA for different anatomical distributions of α-synuclein protein pathology. In AD cohorts, the amygdala-predominant pattern is more common than diffuse neocortical or limbic patterns (‘typical’ α-synuclein protein pathology).^39^^,^^40^ Neuropathological validation studies of α-syn SAA have shown near-perfect sensitivity (97–100%)^41^, ^42^, ^43^ for ‘typical’ α-synuclein protein pathology involving diffuse neocortical and limbic brain regions but much lower sensitivities (14–60%) for the amygdala-predominant pattern.^37^^,^^41^, ^42^, ^43^ Specifically in the AD clinical context, an overall sensitivity of 64–79%^37^^,^^43^ has been reported, with sensitivities based on anatomical distribution at 100% for diffuse neocortical, 57% for transitional limbic and 60% amygdala-predominant.^37^ The specificity for pure AD is reported at 94–100%^44^^,^^45^ with a small number of false positives identified. These findings underscore the importance of understanding the limitations of α-syn SAA in the AD clinical context and how the anatomical distribution of α-synuclein protein pathology may influence its detection.

**Table 1:** Clinical associations of α-syn SAA positivity. Populations not directly comparable due to differing study design.

Study	Quadalti, C. et al. Nature Medicine. 2023^7^	Bellomo, G. et al. Alzheimer’s and Dementia. 2024^6^	Pilotto, A. et al. Alzheimer’s & Dementia. 2023^35^	Collij, L.E. et al. Nature Communications. 2024.^36^	Tosun, D. et al. Alzheimer’s and Dementia. 2024^37^	Silva-Rodríguez, J. et al. Brain 2025^38^
Sample Size	883^a^	240^c^	80^d^	795^e^	1638^f^	865^g^
Study Design (CS, L)	CS, L	CS, L	CS	CS, L	CS, L	CS, L
% SAA positivity	23%^b^	30%	45%	27%^b^	22%^b^	32%^h^
**Clinical associations of α-syn SAA positivity**						
Global cognition	↓	–	–	↓	↓	↓
Memory	↓	–	*NR*	↓	↓	↓
Executive function	↓	−j	*NR*	↓	↓	↓
Language	*NR*^k^	–	*NR*	↓	↓	*NR*
Visuospatial	↓	↓	*NR*	–	–	↓
Cognitive fluctuations	*NR*	*NR*	*NR*	*NR*	*NR*	*NR*
Motor function	↓	*NR*	↓	–	*NR*	*NR*
Hallucinations	↑	−j	↑	*NR*	–	–
RBD symptoms^i^	*NR*	−j^,^^m^	–	*NR*	–	*NR*
Dysautonomia	*NR*	*NR*	↑	*NR*	*NR*	*NR*
Hyposmia	*NR*	*NR*	*NR*	*NR*	*NR*	*NR*
Progression	↑	↑	–	↑	↑^l^	↑

Variations exist within studies with regards to cross-sectional (CS) and longitudinal (L) design, disease stage and kinetic analysis.

NR, Not Reported; ‘–’, No change.

a Memory clinic patients with MCI or dementia.

b Frequency of positivity across entire cohort.

c AD patients, preclinical, prodromal and dementia stages, biomarker-supported.

d AD patients, MCI and mild dementia, biomarker-supported.

e Cognitively impaired individuals (MCI and dementia) derived from the ADNI database.

f Participants included cognitively unimpaired individuals, individuals with MCI, and individuals with clinical diagnosis of dementia due to AD (derived from the ADNI database).

g Individuals with clinical diagnosis of amnestic MCI or AD, derived from the ADNI database.

h Excluding isolated LB pathology.

i Not confirmed with polysomnography.

j Reported difference on kinetic analysis.

k Not reported as individual subdomain.

l Cognitively unimpaired only.

m Night time behaviours on the NPI (Neuropsychiatric Inventory).

Considering this, it is necessary to examine the influence of anatomic distribution of LBP on phenotypic expression. Disease progression modelling of LBD supports distinct spatio-temporal trajectories that associate clinicopathological features. Early limbic disposition is associated with a clinico-pathologic diagnosis of AD, while those with early brainstem/late olfactory bulb deposition demonstrate a greater degree of motor symptoms and hyposmia.^46^ However, there are limited data examining the influence of regional distribution of LBP specifically in the AD clinical context. A small retrospective clinico-pathologic study (n = 33) revealed those harbouring classical (neocortical, limbic, brainstem) LBP were more likely to experience earlier cognitive fluctuations, which could represent an early clinical differentiator for classical LB co-pathology. Several non-significant associations were seen, such as greater frequency of parkinsonism and earlier hallucinations in classical LBP cases and higher frequency of anxiety and apathy in the amygdala-LBP cohort. However, the small sample size may have influenced the statistical power of the study.^47^

Neuropathological studies suggest that the prevalence of ‘typical’ α-synuclein protein pathology increases with age,^22^ while younger cohorts are more likely to exhibit focal amygdala-predominant α-synuclein protein pathology, potentially influencing antemortem CSF α-syn SAA rates.^22^ This pattern appears to hold for α-syn SAA, with two large studies, using data from BioFINDER^7^ and ADNI^37^ finding that α-syn SAA positivity increases with age but to a lesser degree than it does with extent of AD pathology. Furthermore, these studies demonstrate an increase in α-syn SAA with disease stage, in the ADNI cohort from 16% in cognitively unimpaired, to 19% in mild cognitive impairment (MCI) and 38% in dementia, with similarly increasing prevalence noted in the BioFINDER cohort (17% MCI, 21% AD). Taken together, these findings might implicate α-syn SAA positivity as exerting a significant impact on phenotype and clinical trajectory beyond the extent of AD pathology alone.

Clinical identification of α-synuclein co-pathology in AD remains challenging, as characteristic clinical features of LBD are frequently absent in affected individuals. This limits the sensitivity of clinical suspicion for underlying α-synuclein pathology. As such, the need for reliable α-synuclein biomarkers becomes even more critical. Both neuropathological^47^ and α-syn SAA studies^7^ emphasize this, as core clinical features of LBD are often absent in these cohorts. For example, applying clinical criteria for probable DLB (MCI^48^ or dementia stage^49^) and PD^50^ to individuals with LB co-pathology showed that only 9% met clinical criteria at baseline, increasing to 16% during follow-up.^7^ Despite this, α-syn SAA positivity in AD populations exerts significant effects on global cognition, attention, language, executive, visuospatial, and motor functions, with some studies reporting increased prevalence of visual hallucinations.^6^^,^^7^^,^^36^^,^^37^^,^^51^ Data examining the relationship between α-syn SAA and more specific LBD clinical features in AD cohorts, such as anosmia, dysautonomia, and REM sleep behaviour disorder (RBD) remain sparse. Pilloto and colleagues found a slightly higher prevalence of symptomatic orthostatic hypotension (n = 3) in their α-syn SAA positive AD group, with no differences in RBD between the α-syn SAA positive and negative AD groups.^35^ However, the clinical implications of α-synuclein co-pathology extend beyond symptom profiles to disease trajectory. Neuropathologic studies have demonstrated that patients with AD harbouring α-synuclein protein pathology have more severe clinical trajectories over time, with earlier disease onset, faster progression and shorter survival.^40^^,^^52^ A number of α-syn SAA studies have also demonstrated this, with faster decline in global cognition and domain specific deficits over time.^6^^,^^7^^,^^36^, ^37^, ^38^^,^^51^ Data from the ADNI cohort demonstrate greater impairment in global and domain-specific cognitive function (memory, language, executive function) for α-syn SAA positivity MCI-AD and early-AD as compared to their α-syn SAA negative counterparts.^37^ Furthermore, longitudinal analysis of SAA status in AD, demonstrates more rapid decline in α-syn SAA stable positive compared to α-syn SAA stable negative individuals, particularly during the MCI stage.^51^ Collij and colleagues also demonstrated that patients with AD with α-synuclein protein pathology performed worse on cognitive testing at baseline compared to those with isolated AD pathology and demonstrated faster decline in global cognition over time. Interestingly, cognitive decline was not domain-specific in this cohort, despite evidence of more pronounced posterior cortical hypometabolism in those with α-synuclein protein pathology.^36^ Moreover, neuropathological studies in a subgroup of the cohort refuted more severe AD pathology as a feature in those harbouring co-pathology, implying LB pathology exerts an independent effect. The clinical impact of α-synuclein protein pathology in the AD population is further exemplified by its negative impact on cognitive performance in those with low tau burden.^53^ Taken together, these studies suggest that α-syn SAA positivity is crucial to interpret prognostic aspects of cognitive decline, particularly in the earlier phases of the disease.

Therefore, α-syn SAA negative status seems to confer a more innocuous clinical trajectory in AD cohorts. With respect to risk of conversion to α-syn SAA positive status over time, Tosun and colleagues demonstrated a relatively low rate of conversion from α-syn SAA negative to α-syn SAA positive status over time of 5%, with time from last α-syn SAA negative sample to first α-syn SAA positive sample of 2.5 ± 1.6 years on average. The rate of conversion according to baseline diagnostic group was greatest for MCI at 6.7% (cognitively unimpaired (CU) 3.7%, dementia 5.1%).^51^ Supporting this trend, data from an autosomal dominant AD (ADAD) cohort showed no α-syn SAA positivity in presymptomatic patients, with rates increasing to 11% in the symptomatic stage.^54^ These findings highlight the need for longitudinal studies to clarify the temporal dynamics of α-synuclein positivity in AD and future directions are likely to see move from the binary interpretation of the α-syn SAA, to a more nuanced assessment of kinetic parameters^51^ and aggregation status^55^ as potential biomarkers for risk of progression in AD.

While typical late-onset amnestic AD accounts for most clinical cases, the prevalence and characteristics of α-synuclein protein pathology in atypical AD presentations warrant further discussion. As mentioned above, the low sensitivity of α-syn SAA for the amygdala-predominant pattern seen in early-onset AD is reflected in studies of ADAD, where the positivity rate in symptomatic patients is only 11%, despite 83% harbouring α-synuclein protein pathology at autopsy.^54^ Furthermore, the absence of α-syn SAA positivity in asymptomatic ADAD cohorts supports the hypothesis of secondary α-synuclein accrual in AD, particularly in amygdala-predominant variants.^54^ Studies of certain atypical AD presentations, such as posterior cortical atrophy (PCA) and behavioural-dysexecutive variant AD, report higher α-synuclein positivity^6^^,^^35^ whereas lower rates are observed in logopenic variant AD. However, recent neuropathological comparisons between typical and atypical AD presentations did not find a significant association between atypical presentation and α-synuclein protein pathology.^56^ These findings highlight the need for additional research to clarify the role of α-synuclein protein pathology across diverse AD phenotypes.

As the field evolves, the ability of the α-syn SAA to enhance phenotypic profiling and ultimately provide bespoke molecular characterisation of disease in the real-time clinical context is likely to expand, bringing with it significant prognostic and therapeutic implications for patients. As discussed, in its binary form, α-syn SAA requires nuanced interpretation in the AD clinical setting. It also is worth noting the currently limited ability of the assay to distinguish between the synucleinopathies,^44^ with the exception of MSA where conformational differences in the protein affect seeding ability.^57^ However, distinct subtypes of α-synuclein have been shown to exhibit varying morphological and biochemical traits that can influence clinical phenotype and may explain the clinical heterogeneity of this group of disorders.^58^ The impact of cellular *milieu* on strain properties^59^ supports the hypothesis proposed by Just and colleagues that differing cellular environments encountered during the course of spatiotemporal spread impacts the structure and function of the protein.^60^ The influence of cellular *milieu* is of particular interest in AD given the predominance of LBP restricted to the amygdala, which has been hypothesised as pivotal locus for protein misfolding and may play a key role in defining disease trajectory.^61^ Sorrentino and colleagues demonstrated that LBP found in the amygdala of LBD brains is pathologically and biochemically distinct, and more prone to aggregation, than LBP found restricted to the amygdala in amygdala-predominant-AD which may account for the more innocuous trajectory of the latter.^62^

Furthermore, the indisputable impact of co-pathology on disease trajectory requires attention and of relevance to this review, is the relationship between α-synuclein, tau and amyloid β. Differing associations of CSF-based biomarkers highlight the need for further studies in this area. In the ADNI cohort, higher CSF amyloid β burden and lower p-tau181 was linked with higher α-syn SAA positivity rates, particularly at dementia stage,^37^ furthermore SAA conversion was associated with amyloid β pathology and greater cognitive decline.^37^ However, Ding and colleagues found that both aggregated and non-aggregated α-synuclein was more closely associated with both p-tau and t-tau than with amyloid β.^55^ A recent study by Franzmeier and colleagues^63^ demonstrated that CSF α-syn SAA positivity was associated with greater amyloid β-related increases in p-tau181, higher tau-PET burden in AD-typical regions, and faster longitudinal tau accumulation. Additionally, α-synuclein co-pathology was linked to accelerated amyloid β-related cognitive decline, suggesting that α-synuclein may exacerbate amyloid β-driven tau pathology in AD.

The recent addition of CSF α-syn SAA to the criteria for diagnosis and staging of AD^64^ buttresses its role in the diagnostic work-up of neurodegenerative disease. However, the increasing availability of pathologically based biomarkers demands a nuanced interpretation in the clinical setting. Clinical judgement must be exercised when discerning the leading clinical syndrome, notwithstanding the presence of co-pathology.^64^ Furthermore, pertinent to the AD clinical context, is the reduced sensitivity of the α-syn SAA for amygdala-predominant LBP in AD, although the phenotypic influence and therapeutic implications of this subtype remain to be fully elucidated. As the field continues to evolve, greater understanding of strain-specific clinical associations may bring about improved specificity for LBD subtypes and less invasive methods of detecting α-synuclein pathology (such as skin^65^ and blood^66^) could further increase the diagnostic utility of this test. Furthermore, variable morphological and seeding characteristics could see the influence of distinct pathological sites yielding more specific results.

### Progressive supranuclear palsy (PSP) and corticobasal syndrome (CBS)

Diagnosing PSP and CBS in the early stages is challenging due to their significant clinical overlap with other parkinsonian disorders, such as PD and MSA. Although α-synuclein SAAs are highly sensitive and specific for diagnosing PD^2^ and MSA,^3^ neuropathological studies have found that patients with PSP and corticobasal degeneration (CBD) also exhibit α-synuclein protein pathology,^23^^,^^67^^,^^68^ potentially limiting the specificity of α-synuclein SAAs in the differential diagnosis of these diseases.

In response to this, Anastassiadis and colleagues,^69^ Gomes and colleagues^70^ and Vaughan and colleagues^71^ have investigated the prevalence and clinical relevance of α-synuclein co-pathology in atypical parkinsonisms (Table 2). All three research groups discovered that a substantial proportion of patients with PSP and CBS were α-synuclein SAA positive. Anastassiadis and colleagues reported that misfolded α-synuclein was detectable via SAAs in 36% of patients with CBS and 29% of patients with PSP.^69^ Gomes and colleagues reported α-synuclein SAA positivity in 29% of CBD and 25% of PSP^70^ while Vaughan and colleagues identified α-synuclein SAA positivity in 29.7% of patients with CBS and 10.2% of patients with PSP.^71^

**Table 2:** Clinical associations of α-syn SAA positivity in living patients with PSP and CBS.

Study	Sample size	Study design	% α-Syn SAA positivity	Clinical associations of α-Syn SAA positivity
Anastassiadis et al. Neurology. 2024.^69^	67 (CBS: 39, PSP: 28)	Cross-sectional	CBS: 35.9% (14/39) PSP: 28.6% (8/28)	- Lower CSF amyloid β 42 in α-Syn SAA+ - CSF AD biomarker positivity more frequent in α-Syn SAA+ - Higher frequency of RBD symptoms - No significant association with sex, APOE4, or MoCA scores
Vaughan et al., Movement Disorders. 2024.^71^	96 (CBS: 37, PSP: 59)	Longitudinal (PROSPECT-UK cohort)	CBS: 29.7% (11/37) PSP: 10.2% (6/59)	- In PSP: α-Syn SAA + associated with older age, shorter disease duration, more severe motor/cognitive impairment, and higher hyposmia rates - In CBS: α-Syn SAA + SAA + associated with higher rate of AD biomarker positivity (6/9 α-Syn SAA + cases were CBS-AD)

AD, Alzheimer’s disease; APOE4, apolipoprotein E ε4 allele; CBS, corticobasal syndrome; CSF, cerebrospinal fluid; MoCA, Montreal Cognitive Assessment; PSP, progressive supranuclear palsy; RBD, REM sleep behaviour disorder; SAA, seed amplification assay; α-syn, α-synuclein.

Anastassiadis and colleagues further demonstrated a strong association between α-syn SAA positivity and younger disease onset in CBS, as well as a correlation with AD biomarkers, particularly decreased CSF amyloid β levels—suggesting potential interactions among proteinopathies. Moreover, α-synuclein pathology was linked to specific clinical features such as RBD, pointing to its nuanced role in shaping disease presentation. Despite observing lower α-synuclein positivity rates, Vaughan and colleagues suggest that α-synuclein protein pathology may be more common in older patients with PSP,^71^ consistent with the notion that age-related declines in proteostasis contribute to the accumulation of multiple co-pathologies.^23^ Additionally, the authors highlight that, unlike previous clinicopathological studies,^67^^,^^68^ their findings offer preliminary evidence suggesting that α-synuclein co-pathology could exacerbate disease severity and accelerate progression.^71^

Although larger, multicenter studies are needed to fully elucidate the role of α-synuclein co-pathology in these conditions, the three studies converge on the view that a better understanding of α-synuclein’s contribution could improve diagnostic accuracy—enabling distinctions between pure tauopathies and those influenced by α-synuclein co-pathology—and inform personalized therapeutic strategies. They also highlight the population heterogeneity observed across cohorts, which aligns with the variable rates of co-pathologies reported in neuropathological studies of these disorders.^23^^,^^67^^,^^68^ Importantly, these studies also reinforce the trend observed in postmortem analyses showing that α-synuclein co-pathology is more common in patients with CBS who had underlying AD pathology, compared to those with CBD as the primary pathology.

Recognizing α-synuclein co-pathology is thus essential for refining the diagnostic classification of PSP and CBS, and it underscores the need for comprehensive biomarker assessments in atypical parkinsonisms—including 4R-Tau SAAs^72^^,^^73^ —to optimize clinical care and accelerate the development of targeted therapies for these devastating conditions.

### Idiopathic normal pressure hydrocephalus (iNPH)

iNPH is characterised by gait disturbance, cognitive decline, and urinary incontinence, and its hallmark feature is ventriculomegaly, an enlargement of the brain’s ventricles without an apparent cause.^74^^,^^75^ It primarily affects the elderly^76^ and CSF ventriculoperitoneal shunting often alleviates these symptoms.^76^ However, diagnostic challenges arise due to clinical overlaps with neurodegenerative diseases such as AD, PD, and DLB. Studies have shown that co-existing AD-related pathologies, including amyloid β and tau abnormalities, are associated with poorer cognitive outcomes following shunt procedures.^77^, ^78^, ^79^ Furthermore, progressive ventricular enlargement has also been observed in patients with PD^80^ and a handful of neuroimaging studies using dopamine transporter (DAT) imaging have identified striatal dopaminergic deficits in more than 60% of patients with iNPH, which are partially reversed by shunt surgery.^81^^,^^82^

These studies prompted questions about the relationship between α-synuclein protein pathology and iNPH, and the need to distinguish synucleinopathies with ventricular enlargement from iNPH itself. To address these, Giannini and colleagues^83^ Fasano and colleagues^84^ and Weber and colleagues^85^ explored the role of α-synuclein protein pathology and other neurodegenerative biomarkers in iNPH (Table 3). Giannini and colleagues found that approximately 20% of their iNPH cases were positive for α-syn SAA.^83^ This subset of patients exhibited increased parkinsonian signs, specifically greater axial and upper limb rigidity. Similarly, Fasano and colleagues reported the presence of misfolded α-synuclein in 14% of iNPH cases.^84^ While α-synuclein positive patients were clinically similar to α-synuclein-negative patients, they exhibited a wider base of gait at baseline and greater responsiveness to levodopa. Interestingly, Giannini and colleagues also noted that α-syn SAA positive patients more frequently used levodopa and demonstrated a subjective clinical benefit, despite patients with iNPH typically responding poorly to levodopa.^86^

**Table 3:** Clinical associations of α-syn SAA positivity in patients with idiopathic normal pressure hydrocephalus (iNPH).

Study	Sample size	Study design	% α-Syn SAA positivity	Clinical associations of α-Syn SAA positivity
Fasano et al. Annals of Neurology. 2022.^84^	64 iNPH	Cross-sectional	14.0% (7/50 analysed)	- More frequent L-dopa responsiveness - Wider base of support while walking - No difference in MoCA, AD CSF biomarkers or shunt response
Giannini et al. Fluids Barriers CNS. 2022.^83^	293 iNPH	Cross-sectional	20.5% (60/293)	- Higher axial rigidity - Higher upper limb rigidity - Lower MMSE - No differences in AD CSF biomarkers or shunt response
Weber et al. Movement Disorders. 2025.^85^	144 iNPH	Cross-sectional	30.1% (43/143)	- Worse olfactory performance (64% anosmic) - No differences in AD CSF biomarkers

AD, Alzheimer’s disease; CSF, cerebrospinal fluid; iNPH, idiopathic normal pressure hydrocephalus; MMSE, Mini-Mental State Examination; MoCA, Montreal Cognitive Assessment; p-tau, phosphorylated tau; SAA, seed amplification assay; α-syn, α -synuclein.

Weber and colleagues^85^ expanded on these findings by analysing a cohort of 144 iNPH patients, identifying misfolded α-synuclein in approximately 30% of cases using α-syn SAA. These α-syn SAA positive patients showed distinct clinical characteristics, including significant upper limb rigidity, a notably higher prevalence of anosmia (64% compared to 15.3% in α-syn SAA negative patients), and increased hallucinations. Cognitive impairment was slightly more pronounced in α-syn SAA positive individuals, particularly within the subgroup undergoing comprehensive olfactory assessment. Anosmia, a common early symptom of synucleinopathies, was highlighted as a potential clinical marker to identify underlying α-synuclein pathology in iNPH.

Because iNPH predominantly affects older adults - the group at highest risk for age-related changes (e.g., primary age-related tauopathy [PART]) and other protein pathologies^87^^,^^88^ co-pathology is common. In one small cases series 9/9 of patients with clinical NPH had co-pathology with 8/9 having AD and 1/9 having PSP.^87^ Another study in brain biopsies found over 60% had AD co-pathology.^88^ While iNPH remains a distinct clinical-radiological syndrome treatable with a ventriculoperitoneal shunt, emerging evidence underscores the need to screen for AD biomarkers and α-synuclein protein pathology and tailor treatment based on co-pathology profiles, i.e., levodopa trials in α-synuclein positive cases.^83^ Additionally, integrating biomarkers of co-pathology as in the ATN approach,^89^ which aims to provide a biological characterisation of disease may be useful in iNPH to better ascertain the heterogeneity of this disease and improve prognostication after intervention^90^ as there is ample evidence that cognition deteriorates in patient with iNPH^91^, ^92^, ^93^, ^94^, ^95^ and AD co-pathology despite shunting.^90^

### Traumatic brain injuries (TBIs)

TBIs, including mild TBIs (mTBI), have been reported as a risk factor for dementia.^96^ Although the link between repetitive mTBI and chronic traumatic encephalopathy (CTE) has received the most attention, numerous reports have linked a higher risk of neurodegenerative conditions such as AD, PD, and DLB with mTBI.^97^, ^98^, ^99^, ^100^, ^101^, ^102^, ^103^ Numerous studies have also reported the frequent co-pathology of AD, α-synuclein and other proteinopathies with CTE.^103^, ^104^, ^105^, ^106^, ^107^, ^108^, ^109^ Prompted by this association, Vasilevskaya and colleagues used α-syn SAA to investigate α-synuclein protein pathology in former contact sport athletes, who may be at increased risk for α-synuclein-related disorders due to repeated TBIs.^110^ In their study, Vasilevskaya and colleagues examined the presence of misfolded α-synuclein in a cohort of 30 retired athletes and compared the results with healthy controls. They found that six of the athletes (20%) were α-synuclein-positive, including one individual diagnosed with PD. Although demographic and clinical features did not significantly differ between α-synuclein positive and α-synuclein negative athletes, the α-synuclein positive group was, on average, younger and had played contact sports for a longer period.

Further analysis revealed that α-synuclein positive athletes without additional neurological conditions exhibited a significant decrease in grey matter volumes. The areas where this decrease in volume was more evident were right inferior orbitofrontal, right anterior insula, and right olfactory cortices. These findings highlight the importance of studying athletes as a unique cohort for early detection of α-synuclein protein pathology. Furthermore, the presence of misfolded α-synuclein in younger athletes with longer exposure to contact sports underscores the potential link between repetitive TBIs and the early onset of neurodegenerative processes.^110^ However, longitudinal research is necessary to clarify the progression and clinical significance of α-synuclein positivity in this population.

## Implications of co-pathology in disease modifying clinical trials

As previously discussed in this review, while primary proteinopathies define neurodegenerative diseases, it is now well established that co-pathologies are the rule rather than the exception.^5^ This has major implications for disease-modifying clinical trials, which have traditionally focused on single proteins without accounting for additional or overlapping protein pathologies. The recognition that neurodegenerative diseases often involve complex combinations of protein pathologies—including tau, TDP-43, amyloid β, and α-synuclein—sometimes alongside cerebrovascular disease,^5^^,^^22^^,^^23^^,^^111^ has driven a paradigm shift in their clinical characterisation.

A biological staging framework, developed under the auspices of the National Institute on Ageing and Alzheimer’s Association, reconceptualizes AD as a biological process rather than relying solely on syndromic presentation.^89^ This framework addresses co-pathology by expanding the original biomarker classification of A (amyloid), T (tau), and N (neurodegeneration) to include V (vascular) and S (α-synuclein). Similar to precision classification systems used in oncology, this approach is being envisioned for other neurodegenerative diseases.^112^^,^^113^ By incorporating multiple biomarker-defined dimensions, integrated biological and clinical staging acknowledges that common protein pathologies influence the relationship between clinical and biological stages of AD, contributing to heterogeneity in clinical presentation and progression. However, this framework has yet to be translated into interventional clinical trials.

Some reports have already demonstrated that mixed protein pathologies are present in clinical trial participants. A recently published clinicopathologic study identified cerebrovascular and α-synuclein protein pathology in two patients who received AD disease-modifying therapies (DMTs).^114^ Furthermore, CAA is highly prevalent in autopsy series (∼48%).^115^ However, imaging studies significantly underestimate CAA prevalence in patients with AD, detecting only severe cases (∼22%),^115^ as milder pathology remains undetectable *in vivo*. Importantly, amyloid-removing DMTs have been associated with amyloid-related imaging abnormalities (ARIA), which may be partially explained by undetected CAA, a known risk factor for ARIA.^116^

Thus, the recognition of co-existing protein pathologies has substantial implications for the design of DMTs in neurodegenerative diseases. The exact impact of co-pathologies on clinical features remains challenging to quantify, complicating efforts to determine the number of participants required to achieve sufficient statistical power for agents targeting only one pathology (i.e., amyloid β). Stratifying patients by co-pathology could improve precision but would require larger cohorts, further increasing trial costs. Alternatively, precision trials targeting multiple pathologies simultaneously may offer a more effective approach.

In AD, therapies targeting both amyloid β and tau are already being explored in a familial AD trial (Tau NexGen trial). While recent AD trials have demonstrated slowed disease progression with amyloid β removal, the disease continues to progress.^117^^,^^118^ Tau-targeting drugs represent a critical missing link that could further decelerate progression. The Alzheimer’s Tau Platform (ATP) trial is expected to combine anti-amyloid β and anti-tau therapies in prodromal AD.

Considering the growing recognition of co-pathology prevalence in neurodegenerative diseases, it is increasingly clear that DMTs must move beyond single-target approaches. The high prevalence of α-synuclein co-pathology in AD and its significant impact on clinical outcomes strongly support the future integration of anti-α-synuclein therapies into AD treatment paradigms once such agents become available.^6^^,^^119^, ^120^, ^121^ There is growing evidence that α-synuclein might interact with amyloid β and tau, potentially promoting mutual aggregation and worsening neurodegeneration since experimental models have shown that α-synuclein oligomers enhance amyloid β toxicity and tau hyperphosphorylation, driving neuronal dysfunction.^119^, ^120^, ^121^ These findings, largely derived from basic research, suggest that misfolded proteins may facilitate cross-seeding and amplify neurotoxic cascades, however, whether these mechanisms occur in the human brain remains uncertain. Neuropathological studies will be essential to determine the *in vivo* relevance of these interactions and to disentangle the true impact of cross-seeding on disease severity and progression.

In addition to the well-established relevance of α-synuclein and amyloid β pathologies, TDP-43 co-pathology is now recognized as both prevalent and clinically significant. Limbic-predominant TDP-43 pathology^122^ is found in approximately 19–57% of AD cases.^123^^,^^124^ Furthermore, neuroinflammation is now recognized as a central driver of neurodegenerative disease progression. Immune modulation, therefore, represents a rational therapeutic avenue, with the potential to slow or halt neurodegeneration.^125^^,^^126^ Given the complex and overlapping pathologies in most neurodegenerative diseases, the logical next step in disease-modifying therapy is the adoption of combination approaches.^127^, ^128^, ^129^ Combination therapies can simultaneously target multiple pathological proteins (such as amyloid β, tau, α-synuclein, and TDP-43) and modulate neuroinflammation, addressing the multifactorial nature of these disorders.^127^, ^128^, ^129^

## Outstanding questions

As the field moves toward a biological classification of neurodegenerative diseases,^89^^,^^112^^,^^113^ several critical questions remain unanswered. The development of α-syn SAA has transformed the *ante*-*mortem* detection of α-synuclein pathology, however, while α-syn SAA demonstrates high inter-laboratory concordance once standardised,^130^ its implementation involves numerous procedural steps that require meticulous optimization to ensure consistent results across settings. For instance, the assay’s sensitivity to variations in sample preparation and laboratory conditions necessitates careful standardisation to maintain reliability and avoid false positive or negative results.^1^^,^^131^ Additionally, α-syn SAA has limitations in distinguishing disease subtypes, such as diffuse neocortical, limbic transitional, and amygdala-predominant LBD in AD cohorts, which can hinder precise diagnostic differentiation. The assay also lacks robust quantitative capabilities to measure the burden of pathological α-synuclein seeds—meaning the ability to reliably assess the amount pathological protein aggregates in a standardised way—which is critical for tracking disease progression or therapeutic response. Although recent advances, such as the application of microfluidics to SAA platforms, have demonstrated the potential for quantitative readouts, these approaches are still in early stages and require further validation before they can be implemented in clinical settings.^132^ Future research should focus on refining standardised protocols to streamline the assay’s complex workflow and developing quantitative methods to enhance its clinical utility.

To address these challenges, multi-modal biomarker approaches offer significant promise. Skin-based biomarkers have emerged as a less-invasive tool for detecting protein pathologies outside the central nervous system, providing insights into systemic aspects of neurodegenerative diseases. For α-synuclein, studies have identified pathological aggregates in skin biopsies from patients with PD, DLB, and MSA, with high sensitivity and specificity.^65^ These skin-based α-synuclein assays can complement CSF-based α-syn SAA by offering a less invasive diagnostic method, potentially improving early detection and patient stratification. Similarly, skin-based tau-SAAs have shown promise in detecting tau pathology in AD^133^ and distinguishing tauopathies from synucleinopathies.^73^^,^^134^ However, further validation in larger cohorts is needed to establish their diagnostic and prognostic value across diverse neurodegenerative disorders.^135^

Complementary to skin biomarkers, positron emission tomography (PET) imaging with α-synuclein-specific tracers, such as [18F]ACI-12589^136^ and [18F]-F0502B,^137^ could address in the future the limitation of subtype differentiation by visualizing regional differences in α-synuclein deposition.

The independent effects of α-synuclein pathology on cognition also require further exploration. While studies have demonstrated its role in cognitive decline in AD cohorts,^34^^,^^51^ longitudinal analyses are needed to elucidate the temporal dynamics of α-syn SAA positivity and its interplay with co-pathologies like tau and amyloid β, which is crucial for defining α-synuclein’s role in the AD continuum and its impact on anti-amyloid disease-modifying therapies.

Drawing on progress in AD, where α-synuclein and vascular contributions are integrated into staging frameworks, these advancements provide a model for studying co-pathologies in other neurodegenerative diseases. As combination therapies targeting multiple proteinopathies become feasible, biomarkers like α-synuclein and tau in skin and CSF, alongside neuroimaging and genetic analysis, will be essential for stratifying patients, monitoring disease progression, and evaluating therapeutic efficacy. Kovacs and colleagues emphasized the need for biomarkers of pathogenetic processes to complement protein modifications for personalized diagnostic profiles.^18^ By refining α-syn SAA protocols, expanding the use of skin-based biomarkers, and integrating multi-modal approaches, researchers can advance our understanding of neurodegenerative diseases and drive meaningful improvements in patient care.

## Contributors

IMV, MCT, and GGK conceptualized the review. IMV coordinated the writing process by inviting co-authors to contribute sections aligned with their expertise and by collating and editing their input. IMV, SF, SOD, MCT, and GGK contributed to writing the manuscript and performed data curation. IMV prepared Tables 2 and 3; SF prepared Table 1; GGK prepared Fig. 1. All authors read, edited, and approved the final version of the manuscript.

## Declaration of interests

IMV has a pending patent for Diagnostic assays for movement disorders (18/537,455), reports personal fees from Ferrer and receives research funding from the Rossy Family Foundation, the Blidner family, Ajay Virmani and the Michael J. Fox Foundation. SF has received financial support for attending scientific meetings from the NCHD Training Supports Scheme and the Trinity College, School of Medicine. MCT reports funding from the Tanenbaum Institute of Science in Sport, National Institute of Ageing, Canadian Institutes of Health Research, University Health Network Foundation, Weston Brain Foundation and the Michael J. Fox Foundation. She has received consulting fees from Eisai and Eli Lilly and in-kind support from Roche (to institution). She participates as a clinical trial site for studies sponsored by Janssen, Biogen, Avanex, Green Valley, UCB, Novo Nordisk, GSK, BMS, and Passage Bio. She holds unpaid scientific advisory roles with the Women’s Brain Foundation, Brain Injury Canada, and PSP Canada. SOD reports no conflict of interest. GGK reports personal fees from Parexel and Mitsubishi-Tanabe, other funding from the Rossy Family Foundation and the Edmond Safra Foundation. Grants from Krembil Foundation, MSA Coalition, Michael J. Fox Foundation, Parkinson Canada, NIH, Canada Foundation for Innovation, and Ontario Research Fund outside the submitted work; in addition, GGK has a shared patent for 5G4 Synuclein antibody and a pending patent for Diagnostic assays for movement disorders (18/537,455). GGK has also received royalties from Wiley, Cambridge, Taylor Francis, and Elsevier publishers. None of these had influence on this study.

## References

[1] Concha-Marambio L., Pritzkow S., Shahnawaz M., Farris C.M., Soto C. (2023). Seed amplification assay for the detection of pathologic alpha-synuclein aggregates in cerebrospinal fluid. Nat Protoc.

[2] Siderowf A., Concha-Marambio L., Lafontant D.E. (2023). Assessment of heterogeneity among participants in the Parkinson’s progression markers initiative cohort using α-synuclein seed amplification: a cross-sectional study. Lancet Neurol.

[3] Ma Y., Farris C.M., Weber S. (2024). Sensitivity and specificity of a seed amplification assay for diagnosis of multiple system atrophy: a multicentre cohort study. Lancet Neurol.

[4] Forrest S.L., Kovacs G.G. (2025). Current concepts and molecular pathology of neurodegenerative diseases. Pathology.

[5] Forrest S.L., Kovacs G.G. (2023). Current concepts of mixed pathologies in neurodegenerative diseases. Can J Neurol Sci.

[6] Bellomo G., Toja A., Paolini Paoletti F. (2024). Investigating alpha-synuclein co-pathology in Alzheimer’s disease by means of cerebrospinal fluid alpha-synuclein seed amplification assay. Alzheimers Dement.

[7] Quadalti C., Palmqvist S., Hall S. (2023). Clinical effects of Lewy body pathology in cognitively impaired individuals. Nat Med.

[8] Kovacs G.G., Ferrer I., Smith C., Perry A., Kovacs G.G., Jacques T. (2025). Greenfield’s neuropathology.

[9] Yang Y., Shi Y., Schweighauser M. (2022). Structures of α-synuclein filaments from human brains with Lewy pathology. Nature.

[10] Schweighauser M., Shi Y., Tarutani A. (2020). Structures of α-synuclein filaments from multiple system atrophy. Nature.

[11] Yang Y., Garringer H.J., Shi Y. (2023). New SNCA mutation and structures of α-synuclein filaments from juvenile-onset synucleinopathy. Acta Neuropathol.

[12] Enomoto M., Martinez-Valbuena I., Forrest S.L. (2025). Lewy-MSA hybrid fold drives distinct neuronal α-synuclein pathology. Commun Biol.

[13] Martinez-Valbuena I., Visanji N.P., Kim A. (2022). Alpha-synuclein seeding shows a wide heterogeneity in multiple system atrophy. Transl Neurodegener.

[14] Braak H., Del Tredici K., Rub U., de Vos R.A., Jansen Steur E.N., Braak E. (2003). Staging of brain pathology related to sporadic Parkinson’s disease. Neurobiol Aging.

[15] McKeith I.G., Dickson D.W., Lowe J. (2005). Diagnosis and management of dementia with Lewy bodies: third report of the DLB consortium. Neurology.

[16] Attems J., Toledo J.B., Walker L. (2021). Neuropathological consensus criteria for the evaluation of Lewy pathology in post-mortem brains: a multi-centre study. Acta Neuropathol.

[17] Kovacs G.G., Breydo L., Green R. (2014). Intracellular processing of disease-associated alpha-synuclein in the human brain suggests prion-like cell-to-cell spread. Neurobiol Dis.

[18] Trojanowski J.Q., Revesz T., Neuropathology Working Group on MSA (2007). Proposed neuropathological criteria for the post mortem diagnosis of multiple system atrophy. Neuropathol Appl Neurobiol.

[19] Kovacs G.G., Grinberg L.T., Halliday G. (2024). Biomarker-based approach to alpha-Synucleinopathies: lessons from neuropathology. Mov Disord.

[20] Visanji N.P., Lang A.E., Kovacs G.G. (2019). Beyond the synucleinopathies: alpha synuclein as a driving force in neurodegenerative comorbidities. Transl Neurodegener.

[21] Kovacs G.G. (2019). Are comorbidities compatible with a molecular pathological classification of neurodegenerative diseases?. Curr Opin Neurol.

[22] Spina S., La Joie R., Petersen C. (2021). Comorbid neuropathological diagnoses in early versus late-onset Alzheimer’s disease. Brain.

[23] Robinson J.L., Lee E.B., Xie S.X. (2018). Neurodegenerative disease concomitant proteinopathies are prevalent, age-related and APOE4-associated. Brain.

[24] Forrest S.L., Crockford D.R., Sizemova A. (2019). Coexisting Lewy body disease and clinical parkinsonism in frontotemporal lobar degeneration. Neurology.

[25] Forrest S.L., Kril J.J., Kovacs G.G. (2021). Association between globular glial tauopathies and frontotemporal dementia-expanding the spectrum of gliocentric disorders: a review. JAMA Neurol.

[26] Grau-Rivera O., Gelpi E., Nos C. (2015). Clinicopathological correlations and concomitant pathologies in rapidly progressive dementia: a brain bank series. Neurodegener Dis.

[27] Vital A., Fernagut P.O., Canron M.H. (2009). The nigrostriatal pathway in Creutzfeldt-Jakob disease. J Neuropathol Exp Neurol.

[28] Sekiya H., Koga S., Murakami A. (2024). Frequency of comorbid pathologies and their clinical impact in multiple system atrophy. Mov Disord.

[29] Elobeid A., Libard S., Leino M., Popova S.N., Alafuzoff I. (2016). Altered proteins in the aging brain. J Neuropathol Exp Neurol.

[30] Markesbery W.R., Jicha G.A., Liu H., Schmitt F.A. (2009). Lewy body pathology in normal elderly subjects. J Neuropathol Exp Neurol.

[31] Brettschneider J., Irwin D.J., Boluda S. (2017). Progression of alpha-synuclein pathology in multiple system atrophy of the cerebellar type. Neuropathol Appl Neurobiol.

[32] Kovacs G.G., Milenkovic I., Wohrer A. (2013). Non-Alzheimer neurodegenerative pathologies and their combinations are more frequent than commonly believed in the elderly brain: a community-based autopsy series. Acta Neuropathol.

[33] Kim A., Martinez-Valbuena I., Danics K., Forrest S.L., Kovacs G.G. (2025). Contribution of α-synuclein cytopathologies to distinct seeding of misfolded α-synuclein. Brain Pathol.

[34] Livingston G., Huntley J., Liu K.Y. (2024). Dementia prevention, intervention, and care: 2024 report of the lancet standing commission. Lancet.

[35] Pilotto A., Bongianni M., Tirloni C., Galli A., Padovani A., Zanusso G. (2023). CSF alpha-synuclein aggregates by seed amplification and clinical presentation of AD. Alzheimers Dement.

[36] Collij L.E., Mastenbroek S.E., Mattsson-Carlgren N. (2024). Lewy body pathology exacerbates brain hypometabolism and cognitive decline in Alzheimer’s disease. Nat Commun.

[37] Tosun D., Hausle Z., Iwaki H. (2024). A cross-sectional study of α-synuclein seed amplification assay in Alzheimer’s disease neuroimaging initiative: prevalence and associations with Alzheimer’s disease biomarkers and cognitive function. Alzheimers Dement.

[38] Silva-Rodríguez J., Labrador-Espinosa M.A., Zhang L. (2025). The effect of Lewy body (co-)pathology on the clinical and imaging phenotype of amnestic patients. Brain.

[39] Hamilton R.L. (2000). Lewy bodies in Alzheimer’s disease: a neuropathological review of 145 cases using alpha-synuclein immunohistochemistry. Brain Pathol.

[40] Chung E.J., Babulal G.M., Monsell S.E., Cairns N.J., Roe C.M., Morris J.C. (2015). Clinical features of Alzheimer disease with and without Lewy bodies. JAMA Neurol.

[41] Samudra N., Fischer D.L., Lenio S. (2024). Clinicopathological correlation of cerebrospinal fluid alpha-synuclein seed amplification assay in a behavioral neurology autopsy cohort. Alzheimers Dement.

[42] Arnold M.R., Coughlin D.G., Brumbach B.H. (2022). α-Synuclein seed amplification in CSF and brain from patients with different brain distributions of pathological α-Synuclein in the context of Co-Pathology and Non-LBD diagnoses. Ann Neurol.

[43] Hall S., Orrù C.D., Serrano G.E. (2022). Performance of αSynuclein RT-QuIC in relation to neuropathological staging of Lewy body disease. Acta Neuropathol Commun.

[44] Rossi M., Candelise N., Baiardi S. (2020). Ultrasensitive RT-QuIC assay with high sensitivity and specificity for Lewy body-associated synucleinopathies. Acta Neuropathol.

[45] Fairfoul G., McGuire L.I., Pal S. (2016). Alpha-synuclein RT-QuIC in the CSF of patients with alpha-synucleinopathies. Ann Clin Transl Neurol.

[46] Mastenbroek S.E., Vogel J.W., Collij L.E. (2024). Disease progression modelling reveals heterogeneity in trajectories of Lewy-type α-synuclein pathology. Nat Commun.

[47] Roudil J., Deramecourt V., Dufournet B. (2018). Influence of Lewy pathology on Alzheimer’s disease phenotype: a retrospective clinico-pathological study. J Alzheimers Dis.

[48] McKeith I.G., Boeve B.F., Dickson D.W. (2017). Diagnosis and management of dementia with Lewy bodies: fourth consensus report of the DLB consortium. Neurology.

[49] McKeith I.G., Ferman T.J., Thomas A.J. (2020). Research criteria for the diagnosis of prodromal dementia with Lewy bodies. Neurology.

[50] Postuma R.B., Berg D., Stern M. (2015). MDS clinical diagnostic criteria for Parkinson’s disease. Mov Disord.

[51] Tosun D., Hausle Z., Thropp P. (2024). Association of CSF α-synuclein seed amplification assay positivity with disease progression and cognitive decline: a longitudinal Alzheimer’s disease neuroimaging initiative study. Alzheimers Dement.

[52] Brenowitz W.D., Hubbard R.A., Keene C.D. (2017). Mixed neuropathologies and associations with domain-specific cognitive decline. Neurology.

[53] Landau S.M., Lee J., Murphy A. (2024). Individuals with Alzheimer’s disease and low tau burden: characteristics and implications. Alzheimers Dement.

[54] Levin J., Baiardi S., Quadalti C. (2024). α-Synuclein seed amplification assay detects Lewy body co-pathology in autosomal dominant Alzheimer’s disease late in the disease course and dependent on Lewy pathology burden. Alzheimers Dement.

[55] Ding Y., Wang L., Liu J., Deng Y., Jiao Y., Zhao A. (2025). Distinct CSF α-synuclein aggregation profiles associated with Alzheimer’s disease phenotypes and MCI-to-AD conversion. J Prev Alzheimers Dis.

[56] Pina-Escudero S.D., La Joie R., Spina S. (2024). Comorbid neuropathology and atypical presentation of Alzheimer’s disease. Alzheimers Dement (Amst).

[57] Shahnawaz M., Mukherjee A., Pritzkow S. (2020). Discriminating α-synuclein strains in Parkinson’s disease and multiple system atrophy. Nature.

[58] Malfertheiner K., Stefanova N., Heras-Garvin A. (2021). The concept of α-Synuclein strains and how different conformations may explain distinct neurodegenerative disorders. Front Neurol.

[59] Holec S.A.M., Woerman A.L. (2021). Evidence of distinct α-synuclein strains underlying disease heterogeneity. Acta Neuropathol.

[60] Just M.K., Gram H., Theologidis V. (2022). Alpha-synuclein strain variability in body-first and brain-first synucleinopathies. Front Aging Neurosci.

[61] Nelson P.T., Abner E.L., Patel E. (2018). The amygdala as a locus of pathologic misfolding in neurodegenerative diseases. J Neuropathol Exp Neurol.

[62] Sorrentino Z.A., Goodwin M.S., Riffe C.J. (2019). Unique α-synuclein pathology within the amygdala in Lewy body dementia: implications for disease initiation and progression. Acta Neuropathol Commun.

[63] Franzmeier N., Roemer-Cassiano S.N., Bernhardt A.M. (2025). Alpha synuclein co-pathology is associated with accelerated amyloid-driven tau accumulation in Alzheimer’s disease. Mol Neurodegener.

[64] Jack C.R., Andrews S.J., Beach T.G. (2024). Revised criteria for the diagnosis and staging of Alzheimer’s disease. Nat Med.

[65] Zhao Y., Luan M., Liu J. (2025). Skin α-synuclein assays in diagnosing Parkinson’s disease: a systematic review and meta-analysis. J Neurol.

[66] Okuzumi A., Hatano T., Matsumoto G. (2023). Propagative α-synuclein seeds as serum biomarkers for synucleinopathies. Nat Med.

[67] Robinson J.L., Yan N., Caswell C. (2020). Primary tau pathology, not copathology, correlates with clinical symptoms in PSP and CBD. J Neuropathol Exp Neurol.

[68] Jecmenica Lukic M., Kurz C., Respondek G. (2020). Copathology in progressive supranuclear palsy: does it matter?. Mov Disord.

[69] Anastassiadis C., Martinez-Valbuena I., Vasilevskaya A. (2024). CSF α-Synuclein seed amplification assay in patients with atypical parkinsonian disorders. Neurology.

[70] Fernandes Gomes B., Farris C.M., Ma Y. (2023). α-Synuclein seed amplification assay as a diagnostic tool for parkinsonian disorders. Parkinson Relat Disord.

[71] Vaughan D.P., Fumi R., Theilmann Jensen M. (2024). Evaluation of cerebrospinal fluid α-Synuclein seed amplification assay in progressive supranuclear palsy and corticobasal syndrome. Mov Disord.

[72] Saijo E., Metrick M.A., Koga S. (2020). 4-Repeat tau seeds and templating subtypes as brain and CSF biomarkers of frontotemporal lobar degeneration. Acta Neuropathol.

[73] Martinez-Valbuena I., Tartaglia M.C., Fox S.H., Lang A.E., Kovacs G.G. (2024). Four-repeat tau seeding in the skin of patients with progressive supranuclear palsy. JAMA Neurol.

[74] Hakim S., Adams R.D. (1965). The special clinical problem of symptomatic hydrocephalus with normal cerebrospinal fluid pressure. Observations on cerebrospinal fluid hydrodynamics. J Neurol Sci.

[75] Relkin N., Marmarou A., Klinge P., Bergsneider M., Black P.M. (2005). Diagnosing idiopathic normal-pressure hydrocephalus. Neurosurgery.

[76] Jaraj D., Rabiei K., Marlow T., Jensen C., Skoog I., Wikkelsø C. (2014). Prevalence of idiopathic normal-pressure hydrocephalus. Neurology.

[77] Abu-Rumeileh S., Giannini G., Polischi B. (2019). Revisiting the cerebrospinal fluid biomarker profile in idiopathic normal pressure hydrocephalus: the Bologna pro-hydro study. J Alzheimers Dis.

[78] Braun M., Bjurnemark C., Seo W. (2022). Higher levels of neurofilament light chain and total tau in CSF are associated with negative outcome after shunt surgery in patients with normal pressure hydrocephalus. Fluids Barriers CNS.

[79] Lukkarinen H., Jeppsson A., Wikkelsö C. (2022). Cerebrospinal fluid biomarkers that reflect clinical symptoms in idiopathic normal pressure hydrocephalus patients. Fluids Barriers CNS.

[80] Mak E., Su L., Williams G.B. (2017). Longitudinal whole-brain atrophy and ventricular enlargement in nondemented Parkinson’s disease. Neurobiol Aging.

[81] Pozzi N.G., Brumberg J., Todisco M. (2021). Striatal dopamine deficit and motor impairment in idiopathic normal pressure hydrocephalus. Mov Disord.

[82] Todisco M., Zangaglia R., Minafra B. (2021). Clinical outcome and striatal dopaminergic function after shunt surgery in patients with idiopathic normal pressure hydrocephalus. Neurology.

[83] Giannini G., Baiardi S., Dellavalle S. (2022). In vivo assessment of Lewy body and beta-amyloid copathologies in idiopathic normal pressure hydrocephalus: prevalence and associations with clinical features and surgery outcome. Fluids Barriers CNS.

[84] Fasano A., Martinez-Valbuena I., Azevedo P. (2022). Alpha-synuclein RT-QuIC in idiopathic normal pressure hydrocephalus. Ann Neurol.

[85] Weber S., Farris C.M., Ma Y. (2025). Anosmia and upper limb Rigidity-A potential phenotype of idiopathic normal pressure hydrocephalus with cerebrospinal fluid α-Synuclein seeds. Mov Disord.

[86] Molde K., Söderström L., Laurell K. (2017). Parkinsonian symptoms in normal pressure hydrocephalus: a population-based study. J Neurol.

[87] Cabral D., Beach T.G., Vedders L. (2011). Frequency of Alzheimer’s disease pathology at autopsy in patients with clinical normal pressure hydrocephalus. Alzheimers Dement.

[88] Libard S., Alafuzoff I. (2019). Alzheimer’s disease neuropathological change and loss of matrix/neuropil in patients with idiopathic normal pressure hydrocephalus, a model of Alzheimer’s disease. Acta Neuropathol Commun.

[89] Jack C.R., Andrews J.S., Beach T.G. (2024). Revised criteria for diagnosis and staging of Alzheimer’s disease: Alzheimer’s association workgroup. Alzheimers Dement.

[90] Malm J., Graff-Radford N.R., Ishikawa M. (2013). Influence of comorbidities in idiopathic normal pressure hydrocephalus - research and clinical care. A report of the ISHCSF task force on comorbidities in INPH. Fluids Barriers CNS.

[91] Bech-Azeddine R., Høgh P., Juhler M., Gjerris F., Waldemar G. (2007). Idiopathic normal-pressure hydrocephalus: clinical comorbidity correlated with cerebral biopsy findings and outcome of cerebrospinal fluid shunting. J Neurol Neurosurg Psychiatry.

[92] Golomb J., Wisoff J., Miller D.C. (2000). Alzheimer’s disease comorbidity in normal pressure hydrocephalus: prevalence and shunt response. J Neurol Neurosurg Psychiatry.

[93] Leinonen V., Koivisto A.M., Alafuzoff I. (2012). Cortical brain biopsy in long-term prognostication of 468 patients with possible normal pressure hydrocephalus. Neurodegener Dis.

[94] Hamilton R., Patel S., Lee E.B. (2010). Lack of shunt response in suspected idiopathic normal pressure hydrocephalus with Alzheimer disease pathology. Ann Neurol.

[95] Leinonen V., Koivisto A.M., Savolainen S. (2010). Amyloid and tau proteins in cortical brain biopsy and Alzheimer’s disease. Ann Neurol.

[96] Livingston G., Huntley J., Sommerlad A. (2020). Dementia prevention, intervention, and care: 2020 report of the lancet commission. Lancet.

[97] Adams J.W., Alvarez V.E., Mez J. (2018). Lewy body pathology and chronic traumatic encephalopathy associated with contact sports. J Neuropathol Exp Neurol.

[98] Morissette M.P., Prior H.J., Tate R.B., Wade J., Leiter J.R.S. (2020). Associations between concussion and risk of diagnosis of psychological and neurological disorders: a retrospective population-based cohort study. Fam Med Community Health.

[99] Crane P.K., Gibbons L.E., Dams-O’Connor K. (2016). Association of traumatic brain injury with late-life neurodegenerative conditions and neuropathologic findings. JAMA Neurol.

[100] Mackay D.F., Russell E.R., Stewart K., MacLean J.A., Pell J.P., Stewart W. (2019). Neurodegenerative disease mortality among former professional soccer players. N Engl J Med.

[101] Agrawal S., Leurgans S.E., James B.D. (2022). Association of traumatic brain injury with and without loss of consciousness with neuropathologic outcomes in community-dwelling older persons. JAMA Netw Open.

[102] Deutsch M.B., Mendez M.F., Teng E. (2015). Interactions between traumatic brain injury and frontotemporal degeneration. Dement Geriatr Cogn Disord.

[103] Taghdiri F., Khodadadi M., Sadia N. (2024). Unusual combinations of neurodegenerative pathologies with chronic traumatic encephalopathy (CTE) complicates clinical prediction of CTE. Eur J Neurol.

[104] Bieniek K.F., Cairns N.J., Crary J.F. (2021). The second NINDS/NIBIB consensus meeting to define neuropathological criteria for the diagnosis of chronic traumatic encephalopathy. J Neuropathol Exp Neurol.

[105] Asken B.M., Tanner J.A., VandeVrede L. (2022). Multi-modal biomarkers of repetitive head impacts and traumatic encephalopathy syndrome: a clinicopathological case series. J Neurotrauma.

[106] Ling H., Morris H.R., Neal J.W. (2017). Mixed pathologies including chronic traumatic encephalopathy account for dementia in retired association football (soccer) players. Acta Neuropathol.

[107] Mez J., Daneshvar D.H., Kiernan P.T. (2017). Clinicopathological evaluation of chronic traumatic encephalopathy in players of American football. Jama.

[108] Yang C., Nag S., Xing G., Aggarwal N.T., Schneider J.A. (2020). A clinicopathological report of a 93-Year-Old former street boxer with coexistence of chronic traumatic encephalopathy, Alzheimer’s disease, dementia with Lewy bodies, and hippocampal sclerosis with TDP-43 pathology. Front Neurol.

[109] Forrest S.L., Sadia N., Khodadadi M. (2025). Unprecedented combination of rare degenerative pathologies in an octogenarian Ex-Football player. Neuropathology.

[110] Vasilevskaya A., Martinez-Valbuena I., Anastassiadis C. (2023). Misfolded α-Synuclein in cerebrospinal fluid of contact sport athletes. Mov Disord.

[111] Robinson J.L., Richardson H., Xie S.X. (2021). The development and convergence of co-pathologies in Alzheimer’s disease. Brain.

[112] Hoglinger G.U., Adler C.H., Berg D. (2024). A biological classification of Parkinson’s disease: the SynNeurGe research diagnostic criteria. Lancet Neurol.

[113] Simuni T., Chahine L.M., Poston K. (2024). A biological definition of neuronal α-synuclein disease: towards an integrated staging system for research. Lancet Neurol.

[114] VandeVrede L., La Joie R., Horiki S. (2023). Co-pathology may impact outcomes of amyloid-targeting treatments: clinicopathological results from two patients treated with aducanumab. Acta Neuropathol.

[115] Jäkel L., De Kort A.M., Klijn C.J.M., Schreuder F., Verbeek M.M. (2022). Prevalence of cerebral amyloid angiopathy: a systematic review and meta-analysis. Alzheimers Dement.

[116] Sveikata L., Charidimou A., Viswanathan A. (2022). Vessels sing their ARIAs: the role of vascular amyloid in the age of aducanumab. Stroke.

[117] van Dyck C.H., Swanson C.J., Aisen P. (2023). Lecanemab in early Alzheimer’s disease. N Engl J Med.

[118] Sims J.R., Zimmer J.A., Evans C.D. (2023). Donanemab in early symptomatic Alzheimer disease: the TRAILBLAZER-ALZ 2 randomized clinical trial. JAMA.

[119] Shim K.H., Kang M.J., Youn Y.C., An S.S.A., Kim S. (2022). Alpha-synuclein: a pathological factor with Aβ and tau and biomarker in Alzheimer’s disease. Alzheimers Res Ther.

[120] Fouka M., Mavroeidi P., Tsaka G., Xilouri M. (2020). In search of effective treatments targeting α-Synuclein toxicity in synucleinopathies: pros and cons. Front Cell Dev Biol.

[121] Twohig D., Nielsen H.M. (2019). α-synuclein in the pathophysiology of Alzheimer’s disease. Mol Neurodegener.

[122] Nelson P.T., Dickson D.W., Trojanowski J.Q. (2019). Limbic-predominant age-related TDP-43 encephalopathy (LATE): consensus working group report. Brain.

[123] Jo M., Lee S., Jeon Y.M., Kim S., Kwon Y., Kim H.J. (2020). The role of TDP-43 propagation in neurodegenerative diseases: integrating insights from clinical and experimental studies. Exp Mol Med.

[124] Meneses A., Koga S., O’Leary J., Dickson D.W., Bu G., Zhao N. (2021). TDP-43 pathology in Alzheimer’s disease. Mol Neurodegener.

[125] Deczkowska A., Schwartz M. (2018). Targeting neuro-immune communication in neurodegeneration: challenges and opportunities. J Exp Med.

[126] Adamu A., Li S., Gao F., Xue G. (2024). The role of neuroinflammation in neurodegenerative diseases: current understanding and future therapeutic targets. Front Aging Neurosci.

[127] Valera E., Masliah E. (2016). Combination therapies: the next logical step for the treatment of synucleinopathies?. Mov Disord.

[128] Cummings J.L., Tong G., Ballard C. (2019). Treatment combinations for Alzheimer’s disease: current and future pharmacotherapy options. J Alzheimers Dis.

[129] Del Giudice K.P., Cosgaya M., Zaro I. (2024). Anti-alpha synuclein and anti-tau immunotherapies: can a cocktail approach work?. Parkinsonism Relat Disord.

[130] Bräuer S., Weber M., Deuschle C. (2025). High agreement across laboratories between different alpha-synuclein seed amplification protocols. Eur J Neurol.

[131] Fort-Aznar L., Molina-Porcel L., Ramos-Campoy O. (2024). Misfolded α-Synuclein in autosomal dominant Alzheimer’s disease. J Alzheimers Dis.

[132] Gilboa T., Swank Z., Thakur R. (2024). Toward the quantification of α-synuclein aggregates with digital seed amplification assays. Proc Natl Acad Sci USA.

[133] Wang Z., Wu L., Gerasimenko M. (2024). Seeding activity of skin misfolded tau as a biomarker for tauopathies. Mol Neurodegener.

[134] Dellarole I.L., Vacchi E., Ruiz-Barrio I. (2024). Tau seeding activity in skin biopsy differentiates tauopathies from synucleinopathies. NPJ Parkinsons Dis.

[135] Martinez-Valbuena I., Tartaglia M.C., Kovacs G.G., Lang A.E. (2025). Copathology in atypical parkinsonism-the rule rather than the exception?. JAMA Neurol.

[136] Smith R., Capotosti F., Schain M. (2023). The α-synuclein PET tracer [18F] ACI-12589 distinguishes multiple system atrophy from other neurodegenerative diseases. Nat Commun.

[137] Xiang J., Tao Y., Xia Y. (2023). Development of an α-synuclein positron emission tomography tracer for imaging synucleinopathies. Cell.

